# Commentary: Left Ventricular Hypertrophy in Pediatric Hypertension: A Mini Review

**DOI:** 10.3389/fped.2017.00234

**Published:** 2017-10-31

**Authors:** Guillermo A. Perez Fernandez

**Affiliations:** ^1^The Cuban Hospital, Hamad Medical Corporation, Doha, Qatar

**Keywords:** arterial hypertension, left ventricular hypertrophy, early diastolic alterations, prehypertension, tissue Doppler imaging

Woroniecki and colleagues’ article on left ventricular hypertrophy (LVH) in pediatric hypertension (HTN) reviewed current definitions, clinically relevant methods of LVM measurements, and normalization methods as well as its epidemiology, management, and reversibility in children with HTN (1).

Since the publication in 1977 of the First American Task Force for the diagnosis, evaluation and treatment of high blood pressure (BP) in children and adolescents, updated in 1987, 1996, and more comprehensively in 2004 (as Fourth Report), the importance of HTN-related organ damage; in this context, LVH has been widely stressed. Likewise, the latest European Society of HTN guidelines for the management of HTN in children and adolescents published in 2016 has highlighted the assessment of subclinical organ damage as an intermediate stage in the continuum of vascular disease, targeting predominantly LVH (2).

Woroniecki and colleagues’ article (1) exposed the puzzling concern of defining LVH in a pediatric population on the basis of the existence of diverse criteria with pros and cons. These arguments are not new and have contributed to the difficulty in recent years to ascertain a reliable standardization of LVH definition at the early stages of life in children and adolescents suffering from HTN. Although the authors of the article do not mention the connection of prehypertension with LVH, this association exists (3). Also long-established studies, conducted on hypertensive individuals (2), have reported different formulas to define LVH in children and adolescents.

These aspects complicate the resolution of a definition of LVH for prehypertensive patients.

Unquestionably, the search for a definition of LVH in the context of the issues raised in this commentary is complex with potentially multiple answers. To date, no outcome-based solution has been identified. Nevertheless, there is a reasonable expectation that some results will be forthcoming from the American Heart Association’s SHIP-AHOY study, intended to evaluate BP thresholds, ambulatory BP, and metabolic phenotype that predicts hypertensive target organ damage. While SHIP-AHOY will definitely inform future guidelines, currently, after extensive review of 15,000 manuscripts; the new Clinical Practice Guideline for Screening and Management of High Blood Pressure in Children and Adolescents (3) recommends using LVMI but does not address the different measures in the assessment of LVMI, although dictates to perform a first echocardiogram when considering BP medication initiation.

In our opinion, the appraisal of HTN-induced LVH must also include early diastolic alterations as regional mitral Ea, Aa, and the E/Ea ratio by Tissue Doppler Imaging that precede the onset of LVH (4). Those topics are not mentioned in Woroniecki and colleagues’ article (1) and might be key to increasing the consistency of the assessment of HTN-related cardiac organ damage in children and adolescents.

The PESESCAD-HTA study found diastolic abnormalities even in prehypertensive adolescents without LVH, which underlines the relevance of targeting diastolic alterations on individuals prone to be hypertensive in the short term (5).

In short, the issue of LVH in pediatric populations remains challenging. The solution will require large multinational studies to find a more reliable and matching approach to determine the cardiac organ damage that HTN entails in children and adolescents.

## Author Contributions

The author confirms being the sole contributor of this work and approved it for publication.

## Conflict of Interest Statement

The author declares that the research was conducted in the absence of any commercial or financial relationships that could be construed as a potential conflict of interest.
